# A Randomized Controlled Trial of Mindfulness Meditation Combined With BrainLink Intelligent Biofeedback Instrument on Pancreatic Cancer Patients Under Chemotherapy

**DOI:** 10.1002/brb3.70197

**Published:** 2024-12-22

**Authors:** Na Mi, Shu‐Ting Zhang, Xiao‐Li Sun, Ting Li, Yu Liao, Lei Dong, Ling‐Ling Chu

**Affiliations:** ^1^ Department of Hepatobiliary Surgery The Second Affiliated Hospital of Army Medical University Chongqing China; ^2^ Department of Nursing The Second Affiliated Hospital of Army Medical University Chongqing China

**Keywords:** adjuvant chemotherapy, anxiety, meditation, mindfulness, intelligent biofeedback, pancreatic cancer

## Abstract

**Background:**

Adjuvant chemotherapy can promote the 5‐year overall survival rate of pancreatic cancer (PC) patients to 16%–21%. However, the negative emotions of patients, such as anxiety, are usually omitted. Moreover, their disease burden concentrates on pain symptoms, seriously affecting the quality of life of patients. How to improve the negative emotions of PC patients, alleviate their pain, and ameliorate their quality of life has become an urgent issue.

**Objective:**

To explore the effect of mindfulness meditation (MM) combined with the BrainLink intelligent biofeedback instrument on the anxiety, pain, brain fatigue, and quality of life of PC patients under chemotherapy.

**Method:**

A total of 145 PC patients under chemotherapy were admitted to the Department of Hepatobiliary Surgery of the Second Affiliated Hospital of Army Military Medical University from January 2022 to March 2024 and were incorporated as study objects. They were divided into a control group (*n* = 72) and a test group (*n* = 73) according to the random number table. The control group received routine nursing treatment; the experimental group was treated with MM combined with an intelligent biofeedback instrument. The general information, anxiety (SAS), pain (VAS), EEG signal data (degrees of fatigue, concentration, and relaxation and EEG waves), and quality of life (EORTC QLQ‐C30) of the two groups before intervention and 4th, 8th, and 12th weeks after intervention were compared.

**Result:**

There is no significant difference in baseline data and pathological features between the two groups. After the 4th, 8th, and 12th weeks of intervention, the SAS values of the experimental group are remarkably lower than those of the control group (*p *< 0.05). In the 4th, 8th, and 12th weeks of intervention, the VAS scores of the experimental group are apparently lower than those of the control group (*p *< 0.05). In the 4th, 8th, and 12th weeks of intervention, the score of the quality of life (EORTC QLQ‐C30) in the experimental group is significantly higher than that in the control group (*p *< 0.05). In the 4th, 8th, and 12th weeks, compared with the control group, the experimental group patients showed significant improvement in brain fatigue relief, concentration, and relaxation levels (*p *< 0.05), while the experimental group's brain α and θ. The wave showed an upward trend (*p *< 0.05).

**Conclusion:**

MM combined with the BrainLink intelligent biofeedback instrument can significantly mitigate the anxiety, pain severity, and brain fatigue relief degree of PC patients under chemotherapy, thereby promoting their quality of life. It provides a novel intervention for the psychosomatic health of PC patients under chemotherapy.

## Graphical abstract

Mindfulness meditation combined with the BrainLink intelligent biofeedback instrument can significantly mitigate the anxiety, pain severity, and brain fatigue relief degree of pancreatic cancer patients under chemotherapy, thereby promoting their quality of life.

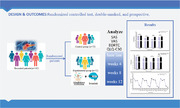

## 1 Introduction

Pancreatic cancer (PC) is a kind of gastrointestinal tumor with high malignancy and unsatisfying prognoses (Cai et al. 2021). The incidence of PC continues to rise, ranking third (He, Schulick, and Del Chiaro 2021) in cancer‐related mortality in the USA and sixth in China (General Office of National Health Commission 2022). Its 5‐year survival rate approximates 10%. Existing medical evidence demonstrates that single‐drug adjuvant chemotherapy can elevate the 5‐year overall survival rate of PC patients treated by surgery alone from 8% to 16%–21%, and further combined chemotherapy can promote their 3‐year overall survival rate to 63.4% (Ghaneh et al. 2023). However, previous research has shown that the incidence of anxiety symptoms in PC patients with therapeutic or palliative cancer treatment is 33%–70% (Davis et al. 2022), significantly higher than other malignant tumors. Negative emotions of patients are usually ignored, which is detrimental to the quality of life of PC patients (Davis et al. 2022; Y. Wang, Bao, and Chen 2023). In addition, plenty of clinical studies on the burden of PC symptoms verify that 90% of PC patients' disease burden focuses on pain symptoms, which significantly deteriorates the quality of life of patients (Chang et al. 2000; Damm et al. 2020; Yang 2023). Therefore, how to improve the negative emotion of anxiety, relieve pain, and improve the quality of life for PC patients under chemotherapy has become an urgent problem.

Mindfulness meditation (MM) is a form of physical and mental intervention constructed by Kabat et al., guiding patients to focus on their current experiences with an open, curious, and accepting attitude (Gok Metin et al. 2019). Modern studies have shown that positive thinking intervention can improve anxiety (Marinelli et al. 2020) and pain (J. F. Liu and Tang 2021) symptoms in PC patients and thus improve their quality of life (Goldberg et al. 2018; Hilton et al. 2017; Wielgosz et al. 2019; Zeidan and Vago 2016). Meanwhile, MM intervention has been applied to breast cancer (Atreya et al. 2018; Bower et al. 2021; Haller et al. 2017; Printz 2021), prostate cancer (Nissen et al. 2020), colon cancer(Atreya et al. 2018), and other cancer populations in a large number of studies, and it can also improve cognitive functioning, as well as depression, insomnia, and other symptoms in cancer patients (Goldberg et al. 2018; Hilton et al. 2017; Wielgosz et al. 2019; Zeidan and Vago 2016). Nevertheless, the outcome measures of MM treatment are mainly subjective scales, lacking measurement and evaluation of objective dimensions.

The BrainLink intelligent biofeedback device is a noninvasive head‐mounted EEG sensor widely used in brain–computer interface systems (S.‐F. Wu, Lu, and Lien 2021). The device displays objective data such as collected EEG signals, relaxation, and concentration in real time (Hilton et al. 2017). The US Federal Communications Commission has licensed the BrainLink device, which has been approved for use in the European Union (CE mark) (Japaridze et al. 2023). Studies have shown that positive thinking meditation affects brain dynamics by influencing attention‐related structures (General Office of National Health Commission 2022). Therefore, the present study combined the Positive Thought Meditation intervention with the BrainLink device: first, to collect data such as EEG signals from Positive Thought Meditation patients in real‐time, objectively, and accurately; and second, to better guide the patients to the Positive Thought Meditation intervention based on the data transmitted by the device to enhance the effect of Positive Thought Meditation on the improvement of pain, anxiety, and other symptoms of patients undergoing chemotherapy for PC.

In summary, this study adopted MM combined with the BrainLink intelligent biofeedback instrument to intervene in the anxiety, pain, and quality of life of PC patients under chemotherapy. Meanwhile, subjective scales and objective EEG monitoring were used as dimensions to evaluate the efficacy of this treatment on PC patients under chemotherapy.

## 2 Method

### 2.1 Study Design and Patient Materials

This study employed trial methods of randomized controlled, double‐masked, and prospective. PC patients who received chemotherapy in the Department of Hepatobiliary Surgery at the Second Affiliated Hospital of Army Medical University (SAHAMU) from January 2022 to March 2024 were selected as study objects. Inclusion criteria were as follows: (1) 18 years old ≤ age ≤ 75 years old; average language expression ability, able to communicate effectively; (2) tumor treated with radical resection surgery, confirmed as PC by pathology; (3) blood routine, liver and kidney function, and electrocardiogram are normal before treatment; the tumor was treated by radical resection, which was proved to be PC by pathology; (4) referring to “Pancreatic Cancer Diagnostic and Treatment Guidelines (2022 Edition)” (General Office of National Health Commission 2022), first‐line chemotherapy regimens are selected according to the patient's physical status, including gemcitabine‐based two‐agent combination regimen, or three‐agent combination FOLFIRINOX or mFOLFIRINOX regimen; (5) patients and their families voluntarily participate in this study, sign the informed consent, have good compliance, and can follow up. Exclusion criteria were as follows: (1) Combined with severe diseases of the heart, liver, kidney, and other organs; (2) Those with previous or combined other malignant tumors; (3) Those with mental diseases or consciousness disorders; and (4) those with severe infections that are difficult to control. Patients who withdraw due to irresistible factors or serious complications are not suitable for continuing the study after being confirmed by a competent physician. The basic information, disease characteristics, intervention measures, disease outcomes, medical expenses, spouse, family, and other information of patients were retrieved from the HIS system. This research was reviewed and approved by the Medical Ethics Committee of the Second Affiliated Hospital of Army Medical University, PLA (Ethics Number: 2021‐Research 161‐03). All patients signed the informed consent form.

### 2.2 Random Assignment and Blinding

The principal investigator numbered the patients in the order of admission, copied 150 numbers from the random number table in order, and copied the random numbers in sequence corresponding to the patient number, stipulating that odd numbers were included in Group A and even numbers were included in Group B. There were 75 cases in each of the two groups. All the questionnaire data were collected by S.T.‐Z. and Y.L. for assessment, and all the study data were analyzed by T.L.

The study was blinded to the subjects, researchers, and statisticians. After the randomized sequence table was generated, the blinded bottom was administered by a study team member other than the investigator. After all the case report forms were entered into the spreadsheet, the statistician performed the data counts for Groups A and B. The statistical analysis was completed before the blind bottom keeper, X.‐L.S., announced the specific groups of A and B.

### 2.3 Sample Size Estimation Method

The required sample size in the study was analyzed using the pwr package in R. The preset presence of a medium effect size *d* = 0.50 (Jacob 1988), the preset statistical test power 1 − *β* = 0.8, and significance level *α* = 0.05 indicated that a minimum of 64 subjects were required in each group. Considering a 20% sample attrition rate, the required sample size was 154 subjects, with *n*1 = *n*2 = 77 subjects per group.

### 2.4 Standard Management

All participants received periodic chemotherapy in day‐care wards. They completed the Self‐Rating Anxiety Scale (SAS), Digital Pain Intensity Scale, and the Quality of Life Scale of the European Organization for Cancer Research and Treatment online before the study and 4, 8, and 12 weeks after it. The control group received routine care. The experimental group was treated with MM and the BrainLink intelligent biofeedback instrument. The intervention standards involved in this study are shown in Table 1.

**TABLE 1:** Specific intervention methods.

Intervention criteria of control group—routine care	(1) Basic care: intravenous catheter care, chemotherapy‐related complications care, etc. (2) Psychological guidance: introduce the environment of the ward and give comfort and encouragement to the patient's anxiety and fear. (3) Chemotherapy drug guidance: introduce the process of chemotherapy infusion, chemotherapy infusion precautions, and so on. (4) Dietary instruction: Instruct patients to have a reasonable diet, with meat and vegetables, balanced nutrition, and small and frequent meals. (5) Discharge guidance: inform the patient of the following review time, and home medication guidance			
Intervention criteria of experimental group—Treatment with MM combined with BrainLink intelligent biofeedback device based on routine care	BrainLink instrument	The BrainLink instrument used in this study is a wearable, single‐channel headband EEG (Epihunter) detection device manufactured by NeuroSky (C. Zhou et al. 2023). The acquisition location of BrainLink is the prefrontal brain, and the waveforms collected are in the frequency band of 0–100 Hz, with a signal transmission frequency of 1S, an ADC precision of 12‐bit ADC, and a response time of less than 2 ms. The device records the patient's band power values of delta, theta, alpha, beta, and gamma brain waves. The device's ThinkGear AM (TGAM) module processes the brain signals. The output of this module reports the user's brain focus and relaxation, which are measured by the patented eSense biometric algorithm to measure whether the brain is focused or relaxed (M. Li et al. 2021) The BrainLink instrument has been licensed by the US Federal Communications Commission and is approved for use in the European Union (CE mark) (Japaridze et al. 2023). Meanwhile, the University of Wollongong in Australia has shown that NeuroSky's technology is 96% accurate compared to EEG technology used in other expensive devices for medical use and that the frequency distribution of EEG waveforms and parameters such as a person's mental state is consistent across all states. Therefore, it can be proved that the BrainLink product used in this test is a validated instrument		
	Preparation before MM operation	Personnel training: One month before the start of this study, an MM team was established, consisting of 1 head nurse (with the title of deputy head nurse engaged in humanistic nursing research), 11 mindfulness mentors (with the intermediate title involved in mindfulness research), 1 health manager (with the intermediate title engaged in psychological research), 1 pain manager (with the intermediate title engaged in pain specialist nurses), and 1 BrainLink mentor (with the intermediate title involved in psychological research), totaling 15 members. The operators of this study received homogeneous training and passed the assessment, obtaining the qualification for mindfulness training		
		Researcher preparation: The researcher recorded the MM (three‐step relaxation, body scanning, seated meditation) guides and soundtrack for the BrainLink‐8‐min meditation program without any adjustments to ensure consistency of the MM intervention. The researcher (Positive Thinking Instructor) explained to the patient the MM's purpose, method, and effects on the body. The researcher distributed the MM‐guided phrases and soundtrack, specified the MM program for each phase of the patient's MM, and taught the participants and their families the specific implementation of MM		
		Patient preparation: The patient should listen to the guidance and music in a quiet and gently lit environment, adopt a comfortable sitting position, close their eyes, inhale through their nose, and exhale through their mouth using a diaphragm. At the same time, pay attention to the gas flow during breathing. If there are distractions, maintain a peaceful attitude and naturally coexist. MM will be treated once a day for 20 min each time, with a course of treatment lasting 4 weeks. The intervention will last for a total of 12 weeks and three courses		
	MM intervention programs	Before the start of the first chemotherapy infusion of chemotherapeutic agents to the fourth week of chemotherapy	Three‐step relaxation method: listen to the guide and the soundtrack to complete the MM. The first step is to meditate on “I can relax” as you breathe in and “I am relaxed like a light cloud” as you breathe out. The second step is to feel yourself becoming more and more relaxed, meditating on “I can be calm” as you inhale and “I am as calm as a serene lake” as you breathe. The third step is to feel yourself becoming calmer and calmer, keep breathing deeply, and meditate on “I am at peace” as you inhale and “I am at peace like a mountain” as you breathe	In a quiet environment, the patient remains seated or lies down, gently closes their eyes, focuses on the breath, feels the changes in the body during breathing, and relaxes the muscles from the top of the head to the soles of the feet. Keep a calm mind throughout the process
		At the time of the fourth chemotherapy session until the eighth week of chemotherapy	Body scanning: listen to the guide and the soundtrack to complete the MM. The participant imagines a soft light moving from top to bottom, outside to inside, starting with the head in turn, including the face, shoulders, chest, abdomen, buttocks, and legs	The patient lies down, the whole body is relaxed, hands are naturally placed on both sides, eyes are closed, and breathing is slow. The patient knows which parts of the body feel comfortable or tired
		From Week 8 chemotherapy to Week 12 chemotherapy	Sitting meditation: Listening to the guided words and the soundtrack to complete the MM, participants observe their breathing, focus on the present moment, and visualize a beautiful scene through the guided words	The patient sits with the legs straight in front of the body, bends the right leg so that the right foot is placed under the left thigh, bends the left leg so that the left foot is placed under the right thigh, places both hands on both knees, makes awareness or wisdom hand prints, keeps the head, neck, and back upright upwards, closes the eyes, relaxes the arms and bends them slightly
	“BrainLink” intervention program	Before the start of the first chemotherapy infusion of chemotherapy drugs At Week 4, chemotherapy At Week 8, chemotherapy At Week 12, chemotherapy	Concentration training: The patient intends to control the virtual apple; the more concentrated the brain's concentration energy is, the easier the virtual apple floats upwards	The smart headband is Bluetooth‐connected to the “Brain link” app, and the patient wears the smart headband on their head to minimize facial movement
			Relaxation training: The patient intends to control the virtual feather and listen to the sound of the feather floating; the more concentrated the brain relaxation energy is, the easier the virtual feather will float upwards	Bluetooth connects the smart headband to the “Brain Link” app. The patient wears the smart headband, chooses a comfortable sitting position, closes their eyes, takes a deep breath, and relaxes the muscles of the whole body

### 2.5 Statistical Analysis

Data acquisition personnel were uniformly trained, methods were standardized, and specified personnel established and maintained a database. Questionnaires issued by researchers surveyed all enrolled subjects. When questionnaires were returned, researchers checked whether there were any omissions. If so, the missing items were filled out on the spot. The questionnaire's effective recovery rate was 100%. All data were analyzed using the statistical software SPSS 26.0 (IBM Corp, Armonk, NY, USA). Continuous variables were expressed as mean ± standard deviation and categorical variables as frequency and ratio. Group means were compared by independent sample *t*‐test and ratios by chi‐square (*χ* ^2^) test. *p* < 0.05 was considered statistically significant.

## 3 Outcomes

**FIGURE 1:**
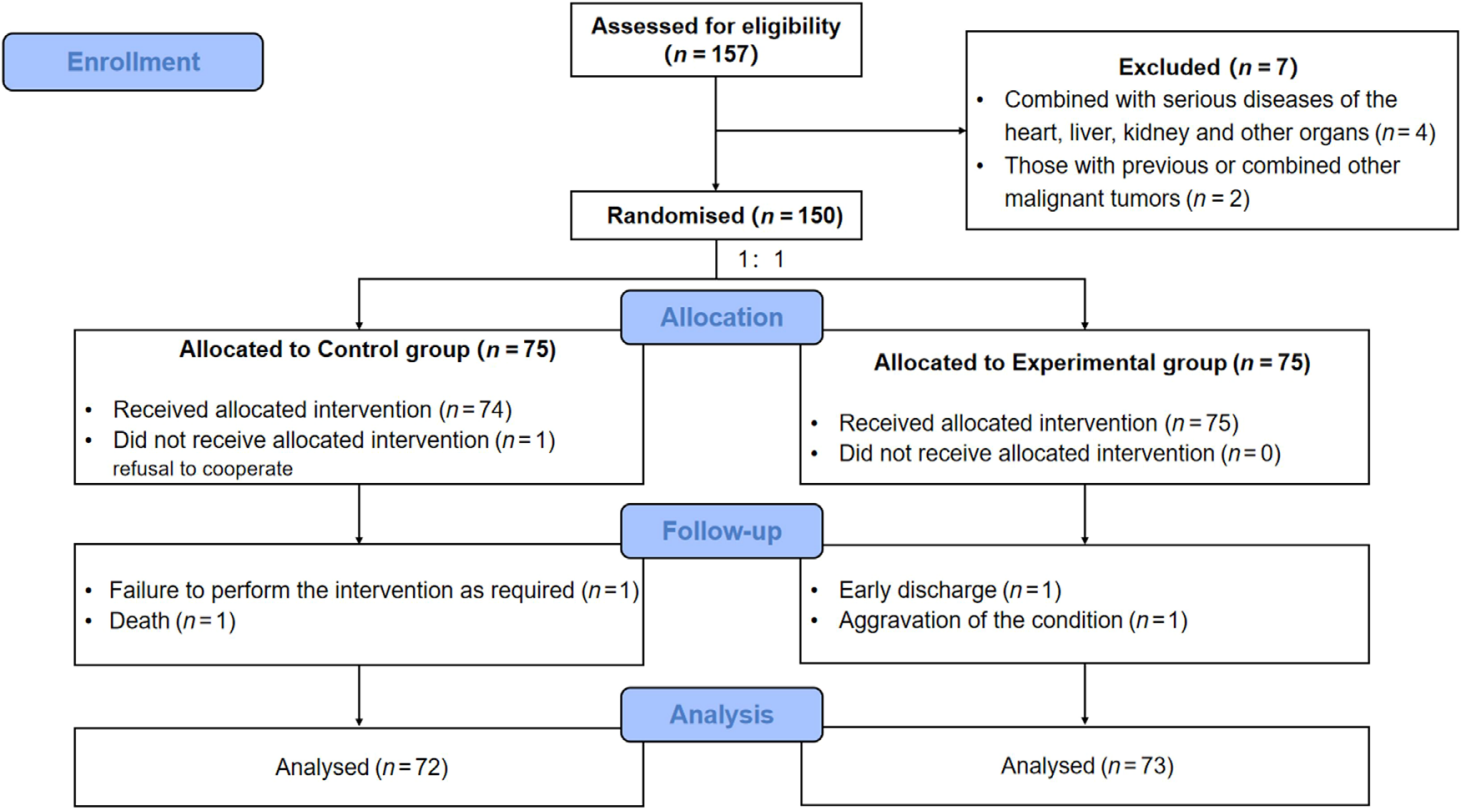
Flowchart of the study design.

In this project, the eligibility of patients was evaluated by N.M. and L.‐L.C. Three cases in the control group were discharged from the study due to failure to comply with the required intervention (*n* = 1), refusal to cooperate (*n* = 1), and death (*n* = 1), respectively; a total of 72 patients in the control group completed all the studies; two cases in the experimental group were discharged from the study due to early discharge (*n* = 1) and worsening of the condition (*n* = 1), respectively; a total of 73 patients in the experimental group completed all the studies. A total of 145 patients finally completed this study (Figure 1).

**FIGURE 2:**
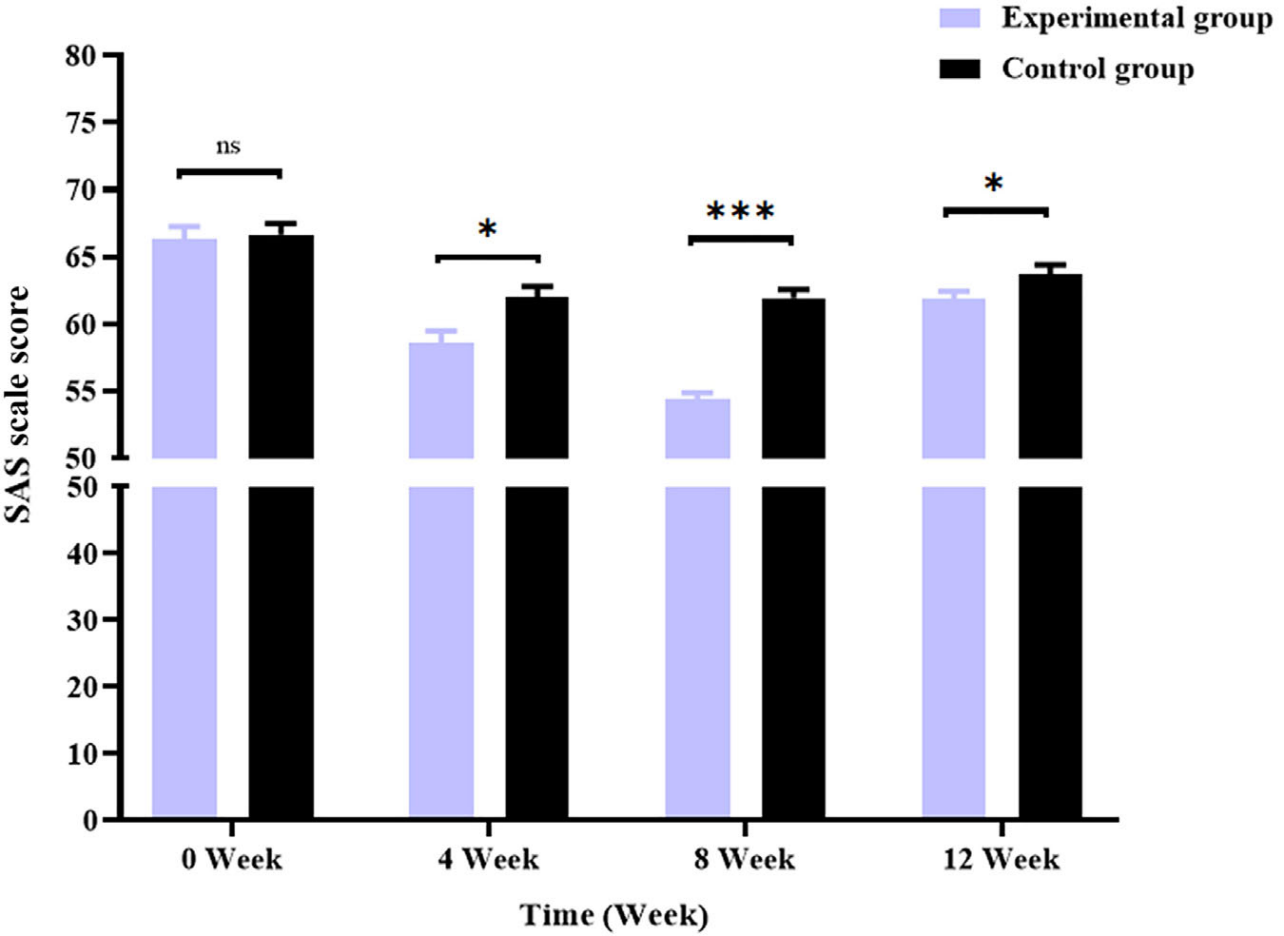
Comparison of SAS scale scores between the two groups before and after intervention. *p < 0.05; **p < 0.01; ***p < 0.001.

**FIGURE 3:**
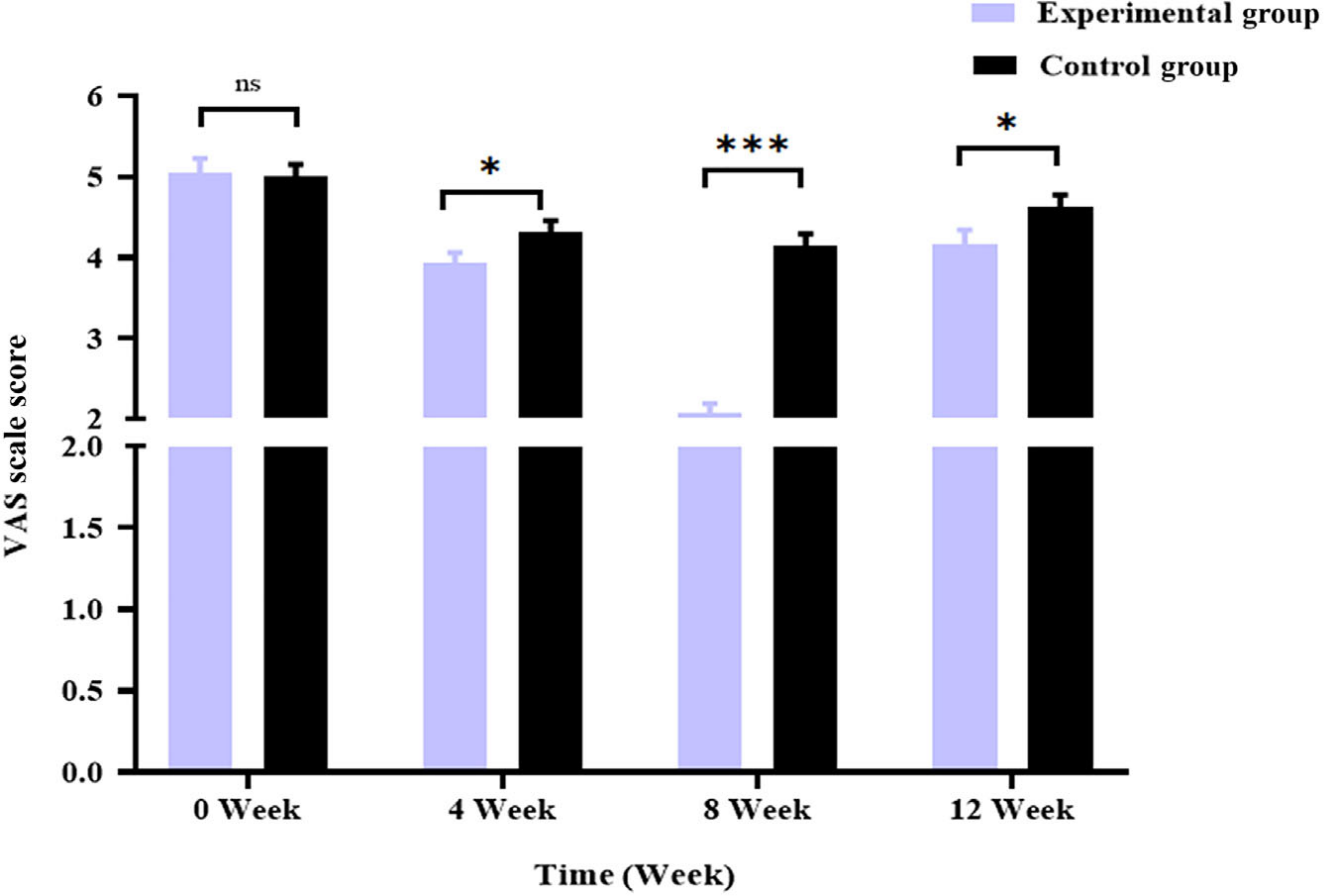
Comparison of VAS scale scores between the two groups before and after intervention. *p < 0.05; **p < 0.01; ***p < 0.001.

**FIGURE 4:**
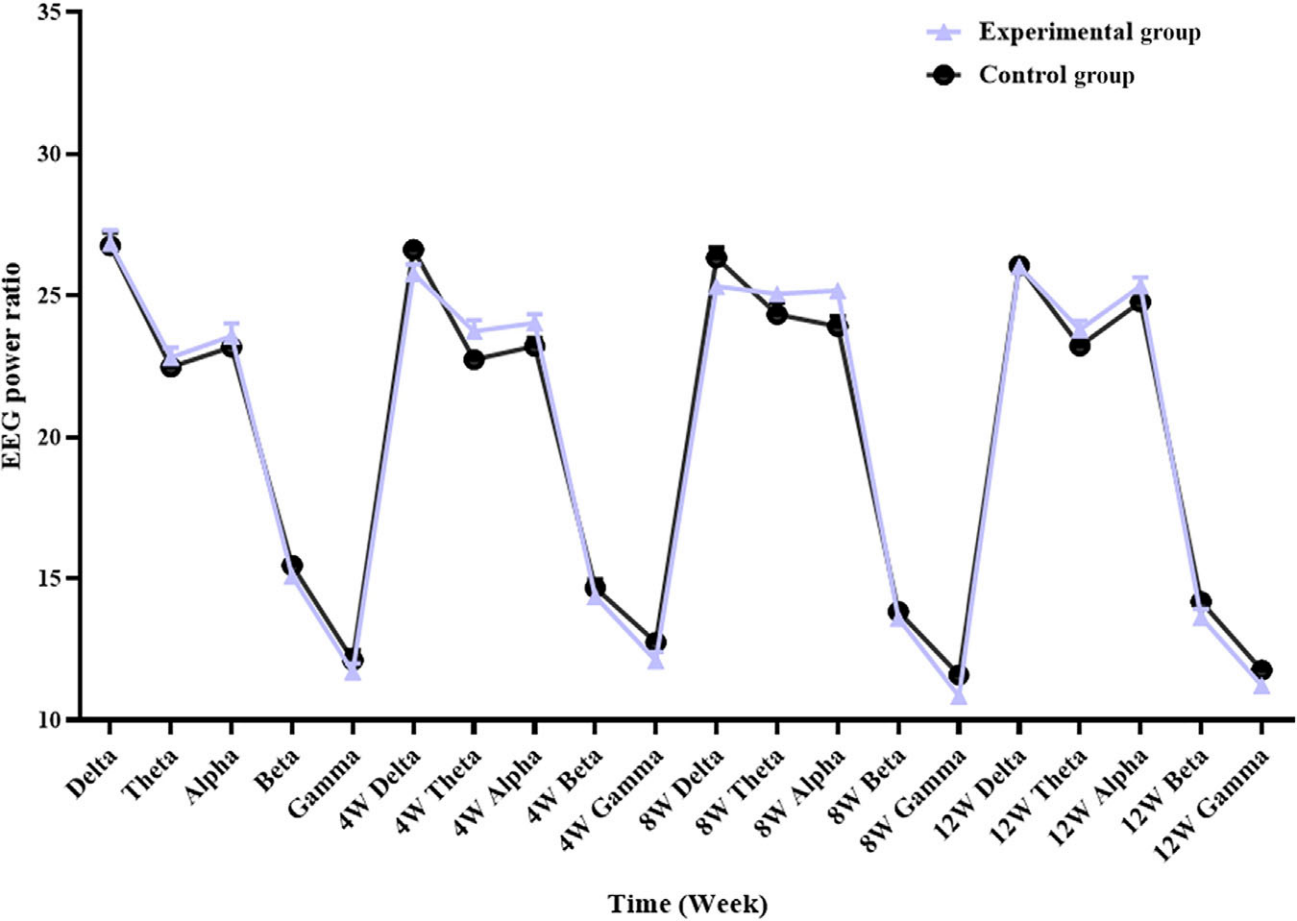
Comparison of the proportion of EEG waves between two groups of patients before and after intervention.

This study evaluated the general information, anxiety, pain, and quality of life of patients using a self‐made general information table (Table 2), the latest version of the SAS (van den Beuken‐van Everdingen et al. 2016) (Table 3; Figure 2), the Visual Analogue Scale (VAS) (van den Beuken‐van Everdingen et al. 2016) (Table 4; Figure 3), and the European Organization for Research and Treatment of Cancer Quality of Life Questionnaire‐Core 30 (EORTC QLQ‐C30) (van den Beuken‐van Everdingen et al. 2016) (Table 5). Monitor the patient's EEG signals through BrainLink to evaluate the patient's EEG activity based on relaxation, fatigue, focus (Table 6), and EEG power ratio (Table 7; Figure 4).

**TABLE 2:** General information table.

Item		Control group (*n* = 72)	Experimental group (*n* = 73)	*x* ^2^/*F*	*p*
Sex	Male	46.00 (63.90)	44.00 (60.30)	0.201	0.654
	Female	26.00 (36.1)	29.00 (39.7)		
Age (years)	18–44	8.00 (11.1)	14.00 (19.2)	2.639	0.267
	45–59	44.00 (61.1)	36.00 (49.3)		
	60–74	20.00 (27.8)	23.00 (31.5)		
Education level	Primary school or below	21.00 (29.2)	19.00 (26.0)	2.017	0.569
	Middle and high school	38.00 (52.8)	45.00 (61.6)		
	Technical and further education	8.00 (11.1)	4.00 (5.5)		
	Undergraduate degree or above	5.00 (6.9)	5.00 (6.8)		
Occupation	Farmer	36.00 (50.0)	33.00 (45.2)	1.636	0.651
	Retired	16.00 (22.2)	17.00 (23.3)		
	Full‐time employment	7.00 (9.7)	12.00 (16.4)		
	Other	13.00 (18.1)	11.00 (15.1)		
Household monthly income	> 1000 Yuan	20.00 (27.8)	22.00 (30.1)	0.191	0.979
	≥ 1000 Yuan	17.00 (23.6)	16.00 (21.9)		
	≥ 3000 Yuan	26.00 (36.1)	25.00 (34.2)		
	≥ 5000 Yuan	9.00 (12.5)	10.00 (13.7)		
Spouse	Yes	61.00 (84.7)	65.00 (89.0)	0.594	0.441
	No	11.00 (15.3)	8.00 (11.0)		
Religious belief	Yes	1.00 (1.4)	3.00 (4.1)	1.000	0.317
	No	71.00 (98.6)	70.00 (95.9)		
Number of offspring	0	2.00 (2.8)	0.00	2.346	0.504
	1	36.00 (50.0)	36.00 (49.3)		
	2	26.00 (36.1)	30.00 (41.1)		
	3 or more	8.00 (11.1)	7.00 (9.6)		
Type of health insurance	Employee health insurance	31.00 (43.1)	26.00 (35.6)	2.760	0.430
	New rural pension scheme	34.00 (47.2)	42.00 (57.5)		
	Urban medical insurance	2.00 (2.8)	3.00 (4.1)		
	Other	5.00 (6.9)	2.00 (2.7)		
Time after diagnosis	≤ 6 months	37.00 (51.4)	38.00 (52.1)	0.322	0.956
	7–12 months	19.00 (26.4)	18.00 (24.7)		
	13–24 months	8.00 (11.1)	7.00 (9.6)		
	≥ 24 months	8.00 (11.1)	10.00 (13.7)		
Surgeries	Yes	47.00 (65.3)	43.00 (58.9)	0.625	0.429
	No	25.00 (34.7)	30.00 (41.1)		
Recurrence	Yes	12.00 (16.7)	17.00 (23.3)	0.993	0.319
	No	60.00 (83.3)	56.00 (76.7)		

**TABLE 3:** Comparison of SAS scale scores between the two groups of patients before and after intervention [*M* (P_25_, P_75_)].

Time	SAS Scale scores		*Z* value	*p*
	Control group (*n* = 72)	Experimental group (*n* = 73)		
Preintervention	66.25 (60.31, 72.19)	67.50 (61.88, 72.50)	0.271	0.786
4 Weeks postintervention	60.00 (56.25, 67.50)	58.75 (52.50, 63.75)	2.323	0.020
8 Weeks postintervention	61.25 (57.50, 66.25)	52.50 (47.50, 57.50)	7.890	< 0.001
12 Weeks postintervention	64.05 (59.78, 68.32)	62.22 (59.78, 64.66)	2.071	0.038

**TABLE 4:** Comparison of VAS scale scores between the two groups of patients before and after intervention [*M* (P_25_, P_75_)].

Time	VAS Scale scores		*Z* value	*p*
	Control group (*n* = 72)	Experimental group (*n* = 73)		
Preintervention	5.00 (4.00, 6.00)	5.00 (4.00, 6.00)	0.043	0.966
4 Weeks postintervention	4.00 (4.00, 5.00)	4.00 (3.00, 5.00)	2.243	0.025
8 Weeks postintervention	4.00 (3.00, 5.00)	2.00 (2.00, 3.00)	8.380	< 0.001
12 Weeks postintervention	5.00 (4.00, 5.00)	4.00 (3.00, 5.00)	2.123	0.034

**TABLE 5:** Comparison of QLQ‐C30 scale scores between the two groups of patients before and after intervention ($\bar{X}+S$).

Time	Item	QLQ‐C30 Scale scores		*t* value	*p*
		Control group (*n* = 72)	Experimental group (*n* = 73)		
Preintervention	Functional scales	94.52 ± 2.88	94.33 ± 2.60	0.413	0.680
	Global health status (QoL)	40.62 ± 17.85	40.64 ± 16.61	0.005	0.996
	Symptom scales	5.90 ± 1.64	5.88 ± 1.60	0.069	0.945
4 Weeks postintervention	Functional scales	94.44 ± 2.61	95.27 ± 2.12	2.105	0.037
	Global health status (QoL)	40.51 ± 16.27	48.06 ± 17.32	2.705	0.008
	Symptom scales	6.09 ± 1.47	5.48 ± 1.12	2.851	0.005
8 Weeks postintervention	Functional scales	94.12 ± 2.52	95.96 ± 1.58	5.239	< 0.001
	Global health status (QoL)	40.51 ± 17.03	48.29 ± 16.89	2.761	0.007
	Symptom scales	6.12 ± 1.60	5.24 ± 1.36	3.574	< 0.001
12 Weeks postintervention	Functional scales	94.20 ± 2.57	94.93 ± 1.63	2.059	0.042
	Global health status (QoL)	40.39 ± 20.06	47.49 ± 22.55	2.001	0.047
	Symptom scales	6.17 ± 1.40	5.47 ± 1.46	2.936	0.004

**TABLE 6:** BrainLink EEG signal data before and after intervention in two groups of patients ($\bar{X}+S$).

Electroencephalogram (EEG) data	Time	BrainLink‐brain fatigue		*t* value	*p*
		Control group (*n* = 72)	Experimental group (*n* = 73)		
Fatigue relief level	Preintervention	17.28 ± 5.73	18.85 ± 6.72	1.515	0.132
	4 Weeks postintervention	19.29 ± 5.13	21.70 ± 7.27	2.305	0.023
	8 Weeks postintervention	18.10 ± 5.78	25.00 ± 6.57	6.718	< 0.001
	12 Weeks postintervention	19.31 ± 5.23	21.30 ± 6.38	2.061	0.041
Concentration level	Preintervention	29.84 ± 3.74	30.01 ± 4.31	0.247	0.805
	4 Weeks postintervention	30.18 ± 4.36	32.38 ± 7.61	2.136	0.035
	8 Weeks postintervention	29.96 ± 3.04	35.06 ± 3.46	9.420	< 0.001
	12 Weeks postintervention	30.57 ± 5.82	32.22 ± 3.73	2.032	0.044
Relaxation level	Preintervention	40.19 ± 6.02	40.74 ± 7.48	0.483	0.630
	4 Weeks postintervention	41.01 ± 6.7	43.38 ± 6.62	2.141	0.034
	8 Weeks postintervention	41.21 ± 5.92	48.44 ± 5.35	7.717	0.001
	12 Weeks postintervention	40.53 ± 9.91	43.99 ± 5.35	2.610	0.010

**TABLE 7:** Comparison of brain wave rate before and after intervention in two groups of patients [*M* (P_25_, P_75_)].

Brain wave	Time	Control group (*n* = 72)	Experimental group (*n* = 73)	*Z* value	*p*
Delta	Preintervention	28.00 (23.73–29.50)	27.10 (23.80–29.25)	0.457	0.648
	4 Weeks postintervention	26.45 (26.00–27.15)	25.90 (24.75–27.60)	1.945	0.052
	8 Weeks postintervention	26.60 (23.73–28.33)	24.80 (24.20–25.50)	1.943	0.052
	12 Weeks postintervention	25.95 (25.28–27.50)	25.80 (25.30–26.80)	0.967	0.333
Theta	Preintervention	22.25 (21.53–22.80)	23.50 (20.55–24.80)	1.920	0.055
	4 Weeks postintervention	23.20 (21.30–24.45)	23.70 (21.55–25.75)	2.164	0.030
	8 Weeks postintervention	24.05 (22.35–25.60)	25.10 (23.50–26.25)	2.332	0.020
	12 Weeks post‐intervention	23.15 (22.30–23.80)	23.90 (21.95–25.85)	1.982	0.047
Alpha	Preintervention	23.10 (22.43–23.60)	24.60 (21.00–26.75)	1.214	0.225
	4 Weeks post‐intervention	23.15 (21.35–24.53)	24.40 (21.90–25.95)	2.245	0.025
	8 Weeks postintervention	24.40 (22.15–26.08)	25.10 (24.30–26.10)	2.213	0.027
	12 Weeks postintervention	24.60 (23.83–25.20)	25.20 (23.70–27.20)	2.191	0.028
Beta	Preintervention	15.25 (14.60–15.80)	14.90 (14.35–15.60)	1.641	0.101
	4 Weeks postintervention	14.75 (12.75–16.60)	14.20 (12.50–16.05)	0.773	0.439
	8 Weeks postintervention	14.00 (13.33–15.00)	13.50 (12.65–14.60)	1.952	0.051
	12 Weeks postintervention	14.35 (13.50–14.98)	13.40 (11.95–15.60)	1.738	0.082
Gamma	Preintervention	11.20 (10.05–14.15)	10.70 (9.85–13.35)	1.203	0.229
	4 Weeks postintervention	12.35 (11.13–14.40)	11.90 (10.15–13.50)	1.954	0.051
	8 Weeks postintervention	11.05 (10.25–12.60)	10.80 (10.30–11.50)	1.962	0.050
	12 Weeks postintervention	11.60 (10.53–12.75)	11.20 (10.05–12.05)	1.883	0.060

## 4 Discussion

PC is a malignant gastrointestinal tumor with increasing incidence and mortality (S. Li et al. 2019). It has meager 5‐year survival rates and poor prognosis. PC patients generally have pain symptoms (van den Beuken‐van Everdingen et al. 2016), which can induce tension and anxiety. Most PC patients need adjuvant treatment, such as chemotherapy and radiotherapy. Chronic pain caused by the tumor, chemotherapy, and radiotherapy will not only torture the patients' physiology but also augment their anxiety (Yue 2023) and decrease their quality of life (Sun et al. 2021; F. F. Zhou 2020). MM is a psychological regulation method extensively applied to alleviate anxiety symptoms. According to a recent meta‐analysis report (Goyal et al. 2014), MM can effectively relieve cancer patients' tension and anxiety and improve their physical symptoms (such as pain). Due to its simple operation and nontoxic side effects, it can be widely used in clinical practice. This study found for the first time that the combination of MM and the BrainLink intelligent biofeedback instrument could alleviate patients' pain and diminish their anxiety, thus advancing their quality of life.

In this study (van den Beuken‐van Everdingen et al. 2016), after 4, 8, and 12 weeks of intervention, the VAS scores of patients in the experimental group are significantly lower than those in the control group (*p* < 0.05). This implies combining MM and the BrainLink intelligent biofeedback instrument can efficiently reduce patient pain. In addition, this study used the BrainLink intelligent biofeedback instrument to monitor the changes in the brain waves of PC patients under chemotherapy. There is a close correlation between the alternation in their pain state and brain electrical activity (especially the α wave and θ wave). The findings of this study are consistent with those of Zeidan (Jensen, Day, and Miró 2014) and Peng (Peng et al. 2020), implying that MM can significantly improve patient pain. The significant reasons are as follows: First, MM can regulate brain transmitters (Y. Liu and Ning 2019; Zhu et al. 2021), trigger pain‐related neuronal responses, and promote sensorimotor α wave, θ wave, and β wave oscillation, thus elevating the pain threshold of patients and diminishing pain severity. Research has revealed a significant correlation between the intensity of pain‐induced θ wave and pain severity. Second, the psychological mechanisms of MM mainly manifest in changes in patients' self‐awareness, awakening state, and sensitivity. MM can transform trainees' awakened state and self‐awareness, altering their sensory sensitivity and lowering the individual perception of pain (J. X. Liu, Lian, and Wang 2022). Meanwhile, the BrainLink intelligent biofeedback instrument can promote patients' concentration and relaxation. The combination of the two enables patients to focus on MM training and transfer their attention away from pain, thereby allaying their accurate perception of pain.

In the 4th, 8th, and 12th weeks of intervention, the SAS scores of the experimental group are remarkably lower than those of the control group (*p* < 0.05). Meanwhile, based on the BrainLink biofeedback instrument, the decreasing rate of brain fatigue relief in the experimental group is higher than in the control group (*p* < 0.05). This demonstrates that the combination of MM and BrainLink biofeedback therapy can profoundly alleviate patient anxiety and fatigue. It agrees with Di Sun et al. (Ren, Zhang, and Jiang 2018; Sun et al. 2021; F. F. Zhou 2020). It may be because MM provides participants with specific perception and imagination through body relaxation, respiration regulation, and attention adjustment (Deng et al. 2023). It inhibits amygdala activity, regulates the autonomic nervous system, enhances the value of α wave, drops the excitability of the sympathetic nervous system, and decreases *β* value, thereby allaying negative emotions such as tension, excitement, and anxiety and downgrading fatigue. This indicates that MM can help patients relax, calm, and actively face cancer (Yan, Huang, and Wu 2018). Second, studies have shown (Zhu, Zhu, and Cai 2016) that among 70%–80% of cancer patients, there is a positive correlation between their pain severity and emotions of anxiety and depression. Research has confirmed (W. Y. Liu 2020) that pain and anxiety share common neurobiological pathways. The hypothalamus–pituitary–adrenal axis (HPA axis) is the most significant among them. By adjusting the neuroendocrine mechanism (R. Wu et al. 2019), MM advances the regulation of the HPA axis and abates stress responses and the level of cortisol (the end product of the HPA axis), thereby further improving anxiety. In addition, it is reported (Frenkel et al. 2023) that MM can lower the degrees of tumor necrosis factor (TNF‐α) and interleukin‐6 (IL‐6), improve inflammation, mediate chronic stress responses, and ease anxiety. This study confirms that when the pain severity of PC patients under chemotherapy drops, their anxiety is significantly improved.

Furthermore, this study is under X. Wang et al. (2020) and Grégoire et al. (2022), indicating that the positive effect of MM on enhancing patients' quality of life strengthens over time. This may be because MM emphasizes conscious attention to the present without judgment and changes trainees' cognition and emotions through training. It encourages patients to understand everything about the disease, alters their attitudes and perceptions toward adverse events, and makes them accept the disease rather than avoid it, thus promoting their mindfulness. This is conducive to improving negative emotions such as anxiety in patients, enabling them to establish a harmonious relationship with pain, elevating their psychological acceptance of pain, abating their perceived severity of pain, and ultimately enhancing their quality of life (Xue et al. 2014).

However, this study merely selected PC patients under chemotherapy from one hospital in Chongqing as the research objects. The sample size is relatively small. Future research can expand the area and sample size to provide a solid scientific basis for the clinical application of MM combined with the BrainLink intelligent biofeedback instrument in improving the chemotherapy treatment of PC patients. Moreover, this study only observed the efficacy in 12 weeks, which is comparatively short. The role of MM combined with the BrainLink intelligent biofeedback instrument in advancing the chemotherapy treatment of PC patients can be further investigated with extended intervention cycles, such as follow‐up in 3, 6, 9, and 12 months, to verify its long‐term efficacy.

## Author Contributions

**Na Mi**: conceptualization, investigation, writing–original draft, writing–review and editing, methodology, formal analysis, resources, data curation, supervision, project administration. **Shu‐Ting Zhang**: conceptualization, investigation, methodology, resources, supervision, formal analysis, validation, software, data curation. **Xiao‐Li Sun**: investigation, project administration, formal analysis, data curation, supervision, resources, validation. **Ting Li**: formal analysis, software, data curation, resources, methodology, writing–original draft, supervision. **Yu Liao**: methodology, software, resources, formal analysis, investigation, conceptualization. **Lei Dong**: conceptualization, methodology, software, data curation, supervision, resources. **Ling‐Ling Chu**: conceptualization, supervision, project administration, writing–original draft, investigation, methodology, data curation, writing–review and editing.

## Ethics Statement

This study has been approved by the Ethics Committee of the Second Affiliated Hospital of the Army Medical University (2021 Study 161‐01) and conducted by the Helsinki Declaration. All patients have signed informed consent forms. Patients can withdraw from the study at any time without explaining the reason. Moreover, the patients should be told they do not need to bear any expenses to participate in this study.

## Conflicts of Interest

The authors declare no conflicts of interest.

### Peer Review

The peer review history for this article is available at https://publons.com/publon/10.1002/brb3.70197.

## Data Availability

Research data are not shared.
